# Opportunities to Improve Primary‐Level Otolaryngology Care: A Survey of Rwandan General Practitioners

**DOI:** 10.1002/oto2.70303

**Published:** 2026-10-17

**Authors:** Kamala Pullakhandam, Christian Kabongo, Mahbuba Tusty, Sarah Nuss, Gabrielle Cahill, Nina Patel, John Bukuru, Hirwa Nteyumwete, Aristote Hakizimana, Aimee Kamaliza, David A. Shaye, Charles Nkurunziza, Eric Uwizeyimana, Ornella Masimbi, Mary Jue Xu, Gratien Tuyishimire

**Affiliations:** ^1^ Harvard Medical School Boston Massachusetts USA; ^2^ Tufts University School of Medicine Boston Massachusetts USA; ^3^ Stanford University School of Medicine Stanford California USA; ^4^ Massachusetts Eye and Ear Boston Massachusetts USA; ^5^ Los Angeles Department of Head and Neck Surgery University of California Los Angeles California USA; ^6^ Loyola Stritch School of Medicine Chicago Illinois USA; ^7^ Rwanda Military Referral and Teaching Hospital Kigali City Rwanda; ^8^ King Faisal Hospital Kigali Rwanda; ^9^ University Teaching Hospital of Kigali Kigali Rwanda; ^10^ Saint Paul's Hospital Millennium Medical College Addis Ababa Ethiopia; ^11^ University of Global Health Equity Kigali Rwanda; ^12^ University of California San Francisco Department of Otolaryngology; ^13^ Department of Otolaryngology University of Rwanda Kigali Rwanda

**Keywords:** education, global health, otolaryngology, primary care

## Abstract

**Objective:**

In Rwanda, 30 OHNS clinicians serve the country's population of 14 million. With low subspecialist density, primary care providers (PCPs) become the first line of otolaryngologic triage and care. However, it is unclear if Rwandan PCPs are equipped to bridge OHNS care gaps.

**Study Design:**

Cross‐sectional qualitative survey‐based study.

**Setting:**

Rwandan PCPs were surveyed, and responses were collected between November 2024 and May.

**Methods:**

Surveys were co‐developed with Rwandan OHNS clinicians and evaluated the diagnostic and management confidence of common head and neck conditions. Responses were analyzed with descriptive statistics.

**Results:**

Forty‐four PCPs completed the survey. PCPs most frequently encountered sinonasal, pediatric, and ear/hearing diseases (mean of 10.5, 9.7, and 6.7 presentations/week, respectively). However, confidence in sinonasal skills was low (mean confidence score of 2.9 out of 5). Diagnostic and management confidence scores were stable across disease categories. Eighty‐eight percent of PCPs identified limited training/knowledge as barriers to patient care. Ninety‐seven percent of PCPs agreed that further emphasis on otolaryngology training is needed during medical school.

**Conclusions:**

This study highlights training gaps and systemic barriers affecting the Rwandan primary care workforce. Targeted hands‐on training is needed to improve PCPs’ skills. Furthermore, evaluation of PCP recognition and management confidence did not reveal specific areas of deficit, indicating that training topics should be comprehensive and diverse. Finally, limited training/knowledge/skills was a common barrier to care, highlighting the importance of PCP‐focused continuing medical education. This survey framework could be utilized in other LMIC settings to identify areas of reduced confidence in otolaryngology provision.

Head and neck conditions comprise a significant proportion of the global burden of disease, disproportionately impacting low and middle‐income countries (LMICs).^1^ Globally, among the 430 million people with disabling hearing loss, 85% reside in LMICs.^2^ And among the 297 million with otitis media, 80% reside in LMICs.^3^ Similarly, LMICs experience 67% of the head and neck cancer burden and over 80% of the mortality.^4^, ^5^

Although LMICs carry a significant proportion of the global otolaryngologic burden, access to otolaryngology subspecialty care remains limited due to workforce shortages. The global density of otolaryngologists is estimated to be 2.19 per 100,000 individuals, with a 57‐fold higher density in high‐income countries than in low‐income countries.^6^ Due to the imbalance between the burden of otolaryngology head and neck disease and the otolaryngology subspecialty workforce, care for these conditions relies heavily on the care provided by the broader primary care workforce. Rwanda is an example of this mismatch between disease burden and workforce. As a low‐income country in central Africa, it has a population of 14 million people and 30 otolaryngologists, for a density of 0.18 otolaryngologists per 100,000 individuals.^6^ Despite a limited number of otolaryngologists, the country has an expanding primary care workforce, with 1300 general practitioners (GPs) operating across its 30 districts, a density of 9.32 GPs for every 100,000 individuals.^7^ Given that the primary care workforce is central to the triage and management of otolaryngologic conditions, evaluating the otolaryngology care delivered at the primary care level—and pathways to enhance it—is a key to improving access and quality at the population level.

We conducted a cross‐sectional survey of GPs in Rwanda to characterize head and neck conditions encountered in clinical practice, self‐reported comfort with management, and barriers to care. With this, we aim to understand the current clinical burden and identify training gaps to inform targeted interventions that strengthen otolaryngology‐specific care in the primary care setting.

## Methods

### Ethics Committee Approval

This study was approved by the Rwanda National Ethics Committee (RNEC413/2024).

### Survey Development

A cross‐sectional survey was developed by Rwandan otolaryngology‐head and neck surgery (OHNS) clinicians in partnership with international collaborators with experience in the Rwandan healthcare system (supplementary materials). The survey was distributed electronically to Rwandan GPs using email and WhatsApp. Survey responses were collected using the REDCap (Research Electronic Data Capture) web‐based platform hosted at the University of Global Health Equity. The survey evaluated three key domains: encountered conditions, confidence in diagnosis and management, and barriers to patient care. Questions on frequency were divided into diseases (weekly estimates) and symptoms (monthly estimates). Monthly symptom frequency responses were categorical (0‐5, 6‐14, 15‐25, and >25) and were converted to ordinal scores (1‐4) for analysis.

Confidence in skills was assessed by disease recognition (31 otolaryngology pathologies), management (25 pathologies), and procedural skills (14 skills). Responses were rated on a Likert scale from 1 (*Not confident*) to 5 (*Very confident*). The survey also included questions addressing barriers to care, including challenges faced by GPs and by patients.

### Survey Distribution

Survey respondents were identified through outreach with the Director Generals of District Hospitals, contact with the University of Global Health Equity, and word of mouth.

Surveys were electronically distributed using the REDCap electronic data capture tools hosted at the University of Global Health Equity. The survey link was first emailed to 10 GPs on November 11, 2024. The survey was also emailed to 36 GPs on November 12, 2024, and they received a survey completion reminder on November 18, 2024. Subsequent recruitment efforts yielded 47 GPs who were later emailed the survey on March 29, 2025. Nine of these GPs had already been invited to fill out the survey in November. In total, 84 unique GPs were invited to complete the survey. No financial or other inducements were attached to survey completion.

### Data Analysis

Survey responses were analyzed using descriptive statistics in Microsoft Excel (Microsoft Corp; Redmond, WA), and Pearson correlation coefficients were calculated using R Statistical Software (R Foundation for Statistical Computing; Vienna, Austria).

## Results

### Demographics

Forty‐four GPs completed the survey, yielding an overall survey response rate of 52% (44/84). The majority of respondents were male (65.9%) (Table 1). The median age of participants was 30 (IQR 27‐33), and the median number of years in practice was 2 (IQR 1‐3). Most GPs practiced in district hospitals (61.4%) and provincial referral hospitals (25%). Surveyed GPs represented 21 of 30 total districts in Rwanda. Many GPs reported having 1 month or less of otolaryngology teaching in medical school (n = 28, 65.9%). Nearly all (97.7%) GPs endorsed the need for further ENT training in GP practice.

**Table 1:** Survey Respondent Demographics

	General practitioners (N = 44)
Gender, n (%)	
Male	29 (65.9)
Female	15 (34.1)
Age, median (IQR)	30 (3)
Years in practice, median (IQR)	2 (1)
Primary practice setting, n (%)	
District hospital	27 (61.4)
Provincial referral hospital	11 (25)
Teaching hospital	3 (6.8)
Health center	2 (4.6)
Private clinic	1 (2.3)
Districts represented	21
ENT exposure in medical school for general practitioners, n (%)	
>2 months	11 (25)
1 to 2 months	1 (2.3)
2 weeks to 1 month	21 (47.7)
<2 weeks	7 (15.9)
None	1 (2.3)
Unknown	3 (6.8)

### Disease Frequency

General practitioners in Rwanda encounter a diverse array of head and neck conditions. The most frequently encountered were sinonasal, pediatric, and ear and hearing conditions, with mean weekly patient presentations of 11 (SD 8.3), 10 (SD 9.6), and 7 (SD 4.5), respectively (Figure 1). The most frequently encountered symptoms were rhinitis/sinusitis, difficulty/noisy breathing, and sore throat, with mean monthly presentation scores of 2.4 (SD 0.1), 2 (SD 0.9), and 1.9 (SD 0.9), respectively.

**Figure 1:**
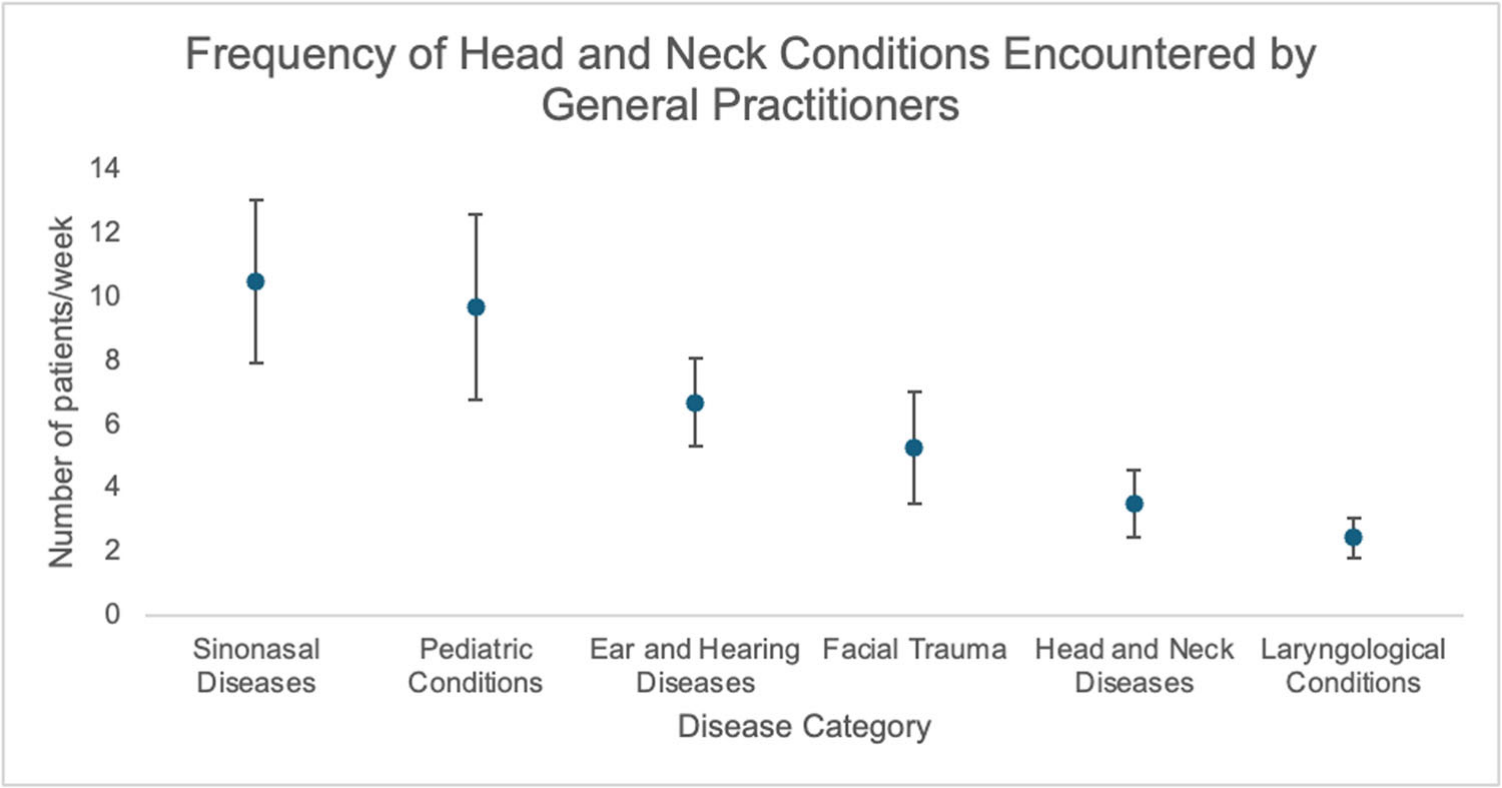
Weekly disease presentation, as reported by general practitioners. Error bars denote 95% confidence intervals.

### Confidence in Disease Recognition, Initial Management, and Procedures

Confidence in recognition and management varied. Among all categories, airway and throat conditions yielded the highest recognition confidence score, with a mean score of 3.4 (SD 1.2) (Table 2). Sinonasal symptom recognition was also high, with a mean score of 3.3 (SD 1.3). The conditions with the lowest mean recognition confidence scores were auricular hematoma/perichondritis, laryngeal cancer, and benign paroxysmal positional vertigo.

**Table 2:** General Practitioner Confidence in Recognition and Management of Otolaryngology Conditions

Condition	Mean recognition confidence (SD)	Mean management confidence (SD)
Ear	3.1 (1.3)	2.9 (1.2)
Wax impaction in ear	4.1 (1)	4 (1)
Otitis externa	3.9 (1)	3.8 (1)
Otitis media	3.9 (1.1)	3.7 (1)
Foreign body in ear	3.8 (1.1)	3.3 (1)
Perforated tympanic membrane	3.4 (1.2)	‐
Mastoiditis	2.9 (1.3)	2.7 (0.9)
Sudden sensorineural hearing loss	2.5 (1.1)	2.3 (0.9)
Malignant otitis externa	2.4 (1)	2.2 (0.9)
Cholesteatoma	2.4 (1.1)	‐
Benign paroxysmal positional vertigo	2.3 (1.1)	2.4 (1.1)
Auricular hematoma/perichondritis	2.2 (1.1)	2 (0.8)
Sinonasal	3.3 (1.3)	3 (1.2)
Epistaxis	4.3 (0.9)	3.7 (1)
Infective and/or allergic rhinitis	3.9 (1.1)	3.8 (0.9)
Foreign body in nose	3.7 (1.2)	3.3 (1.1)
Sinusitis	3.6 (1.1)	3.6 (1)
Choanal atresia	2.8 (1.2)	2.1 (0.9)
Acute orbital complication of rhinosinusitis	2.5 (1.1)	‐
Nasal septal hematoma/abscess	2.4 (0.9)	‐
Orbital cellulitis/abscess	‐	2.8 (0.9)
Nasal septal hematoma/abscess/nasal fracture	‐	2.0 (0.8)
Airway/throat	3.4 (1.2)	3.6 (1)
Tonsillitis	4 (0.9)	3.8 (1)
Gastro‐esophageal reflux disease	3.7 (0.9)	3.7 (1)
Adenoid/tonsillar hypertrophy	3.5 (1.2)	‐
Airway obstruction	3.5 (1.2)	3.2 (0.9)
Pharyngitis/laryngitis	3.4 (1)	3.7 (0.9)
Laryngeal cancer	2.2 (1.1)	‐
Trauma	3.1 (1.2)	2.5 (1)
Foreign body in throat/esophagus	3.3 (1.3)	2.7 (1)
Foreign body in trachea/bronchus	3.1 (1.2)	2.4 (1)
Facial fractures	3 (1.1)	‐
Other/general	2.9 (1.2)	2.4 (1)
Head and neck masses	3.4 (1.1)	‐
Facial nerve palsy/Bell's palsy	3.4 (1.3)	2.6 (1.1)
Ludwig's angina	2.4 (1.1)	2.3 (1)
Deep space neck abscesses	2.4 (1)	2.3 (0.9)

Confidence ranges from 1 (*not confident*) to 5 (*very confident*).

Airway and throat conditions additionally yielded the highest management confidence score, with a mean score of 3.6 (SD 1) (Table 2). Sinonasal disease management was also high, with a mean score of 3 (SD 1.2). The conditions with the lowest mean management confidence scores were auricular hematoma/perichondritis, nasal septal hematoma/abscess/nasal fracture, and choanal atresia. Overall, providers reported higher confidence in disease recognition than in management, with the exception of airway and throat conditions.

Confidence in performing otolaryngology skills varied greatly. Overall, GPs reported the greatest confidence in ear‐related skills, with an average confidence score of 3.3 out of 5 (Table 3). The skills with the highest confidence ratings were otoscopy, ear syringing, and recognition of normal ear, nose, and throat anatomy. Tracheostomy care, anterior rhinoscopy, and lymph node biopsy had the lowest confidence scores. Despite the frequent presentation of sinonasal diseases, there was low confidence in performing anterior rhinoscopy and posterior nasal packing for epistaxis. Pearson correlation coefficient testing demonstrated a significant, positive relationship between years in practice and skill confidence (*r* = 0.4, *p* = .008).

**Table 3:** Confidence in Otolaryngology Skills Reported by Rwandan General Practitioners

Skill	Mean confidence (SD)
Ear	3.3 (1.1)
Otoscopy	3.7 (1.1)
Ear syringing	3.5 (1.1)
Ear foreign body removal	3.2 (1)
Tuning fork test	2.9 (1.2)
Trauma	3.3 (1.1)
Repair of simple facial lacerations	3.3 (1.1)
Sinonasal	2.9 (1.1)
Anterior nasal packing for epistaxis	3.4 (1)
Nasal foreign body removal	3.1 (1)
Posterior nasal packing for epistaxis	2.7 (1)
Anterior rhinoscopy	2.4 (1.1)
Airway/throat	2.1 (1.1)
Tracheostomy care	2.1 (1.1)
Other	2.8 (1.2)
Recognizing normal ear, nose, and throat anatomy	3.8 (1)
Neck examination	3.3 (1)
Incision and drainage of superficial neck abscesses	2.4 (1.1)
Lymph node biopsy	1.8 (1)

Confidence ranges from 1 (*not confident*) to 5 (*very confident*).

### Barriers to High‐Quality Care

GPs frequently identified limited training, knowledge, and skills (88.4% of GPs) in addition to limited equipment and materials (83.7% of GPs) and limited diagnostic tools (74.4% of GPs) as barriers to care (Table 4). Among the care gaps experienced by patients, GPs most frequently saw delayed transfers due to a lack of bed availability at the receiving facility and preventable complications resulting from delayed transfers.

**Table 4:** Barriers and Patient Care Gaps Experienced by General Practitioners

Barrier experienced	General practitioners, n (%)
Limited training/knowledge/skills	39 (88.4)
Limited equipment and materials to treat ENT conditions	37 (83.7)
Limited diagnostic tools to work up ENT diseases	32 (74.4)
Delayed patient presentation	25 (55.8)
Challenges with referring patients to higher level facilities	24 (54.5)

Gaps in patient care and the healthcare system	Frequency (1‐*Never* to 5‐*All the time*)
Delayed transfer due to bed availability at the accident and emergency department?	3.4
Preventable complications resulting from delayed transfer?	3.3
Difficulties in getting an appointment to an ENT specialist?	2.9
Patient/family refusal to transfer to an ENT specialist?	2.7

## Discussion

This survey of GPs in Rwanda identified the most common otolaryngology conditions encountered at the primary care level, confidence of GPs in otolaryngology care, and barriers to care from the primary care perspective. Of the disease categories evaluated, sinonasal diseases were the most frequently encountered and revealed the greatest average recognition confidence among GPs. Despite the frequent presentation of sinonasal pathologies, confidence in performing anterior rhinoscopy and posterior nasal packing for epistaxis was poor. Furthermore, on average, confidence in management and procedure performance was lower than confidence in recognition. Finally, the most frequently reported barriers to providing otolaryngology care from the GP perspective were modifiable–limited training, knowledge, and skills—with nearly all GPs agreeing that medical schools should have increased emphasis on otolaryngology training.

These results suggest that GPs are most comfortable recognizing the conditions they see most frequently, consistent with literature demonstrating an association between case exposure and confidence.^8^, ^9^ Although GPs can recognize common conditions, they report low confidence in otolaryngology skills and limited training and knowledge. This aligns with limited otolaryngology exposure in medical school, as most respondents reported 1 month or less of training during their 5–6 years of medical education, a pattern observed across multiple countries, including high‐income countries such as the United Kingdom and the United States.^10^, ^11^, ^12^ In addition to limited exposure being universal, opportunities to strengthen otolaryngology knowledge among primary care providers also hold true, as multiple studies from the United States show.^13^, ^14^, ^15^

To strengthen otolaryngology knowledge and skills among the primary care workforce, adjunct training during and following medical school can be leveraged. In Rwanda, our team has developed a one‐week simulation‐ and skill‐based otolaryngology curriculum, that was found to improve knowledge and confidence in otolaryngology skills among medical students.^12^ In Zambia, the implementation of dedicated ENT training among healthcare workers—including nurses, clinical officers, and non‐ENT medical doctors—led to improvements in knowledge, attitudes, and practice immediately post‐training as well as five months after the training.^13^ The global emphasis on strengthening the primary care workforce is further reflected in World Health Organization (WHO) policies, including resources such as the Primary Ear and Hearing Care Training Manual to improve otologic care among the primary care workforce.^16^ Reassuringly, in Nigeria, the implementation of an adapted WHO ear and hearing training course improved primary healthcare workers’ ability to recognize risk factors for ear diseases and discourage harmful cultural practices.^17^ Similarly, in Malawi, a 3‐day ear and hearing training using WHO tools improved knowledge and confidence among community healthcare workers.^18^ Overall, there is a clear need to prioritize improvements in global access and quality of otolaryngology care. Investments in further training for primary care providers are not only feasible but have demonstrated high efficacy in achieving this goal.

This study has several limitations. Although there are approximately 1300 GPs currently practicing in Rwanda, our study captured only a subset of practitioners who may not be representative of all GPs across the country. Although this survey was centered on GPs, it is also important to review the perceptions of other primary care providers in Rwanda, including nurses and community health workers. These individuals primarily operate in health centers, which have significantly fewer resources than district hospitals.^19^ Thus, their experiences are likely different from what we have seen among GPs, and it is therefore important that their attitudes are appropriately cataloged as well. Scoping reviews of nurses and community health workers in LMICs have identified insufficient knowledge as a key barrier to care, further emphasizing that these individuals also experience training gaps which limit their practices.^20^, ^21^ However, it is unclear if specific training gaps align with or diverge from those experienced by GPs.

Our study highlighted key areas of intervention to enhance GP otolaryngology practice and confidence. Our findings support a growing body of evidence emphasizing the need to strengthen otolaryngology training in medical school and beyond. Future studies could expand to include nurses and community health workers who also play pivotal roles in the initial management and triage of otolaryngology conditions. Finally, this study is a model for understanding subspeciality knowledge and opportunities for training interventions in subspeciality care more broadly.

## Conclusion

In this cross‐sectional study, we find that limited otolaryngology training, knowledge, and skills contribute to reduced confidence among Rwandan GPs. Shortages in the subspecialist workforce necessitate that primary care providers provide essential otolaryngologic care, but exposure during medical school is limited. Thus, it is critical that structured, longitudinal otolaryngology training opportunities are made available to primary care providers. This comprehensive survey‐based assessment is an effective tool that can be applied in other LMIC settings to identify areas of otolaryngology disease burden and barriers to care among primary care providers.

## Author Contributions

**Kamala Pullakhandam**, study design, study implementation, data collection, data analysis, manuscript drafting and revision; **Christian Kabongo**, data analysis, manuscript drafting and revision; **Mahbuba Tusty**, data analysis, manuscript drafting and revision; **Sarah Nuss**, study conception, study design, manuscript drafting and revision; **Gabrielle Cahill**, data analysis, manuscript drafting and revision; **Nina Patel**, data collection, data analysis, manuscript drafting and revision; **John Bukuru**, study conception, study implementation, manuscript drafting and revision; **Hirwa Nteyumwete**, study conception, study implementation, manuscript drafting and revision; **Aristote Hakizimana**, study conception, study implementation, manuscript drafting and revision; **Aimee Kamaliza**, study conception, study implementation, manuscript drafting and revision; **David A. Shaye**, study conception, study design, manuscript drafting and revision; **Charles Nkurunziza**, study conception, study implementation, manuscript drafting and revision; **Eric Uwizeyimana**, study conception, study implementation, manuscript drafting and revision; **Ornella Masimbi**, study conception, study implementation, manuscript drafting and revision; **Mary Jue Xu**, study conception, study design, study implementation, data collection, data analysis, manuscript drafting and revision; **Gratien Tuyishimire**, Study conception, study design, study implementation, data collection, data analysis, manuscript drafting and revision.

## Disclosures

### Competing interests

None.

### Funding source

None.

## Supporting information

Supporting File 1.

## References

[1] Olusanya BO , Neumann KJ , Saunders JE . The global burden of disabling hearing impairment: a call to action. Bull World Health Organ. 2014;92(5):367‐373. 10.2471/BLT.13.128728 PMC4007124 24839326

[2] World Health Organization . https://www.who.int/news-room/fact-sheets/detail/deafness-and-hearing-loss#:~:text=Over%205%25%20of%20the%20world's,affected%20by%20disabling%20hearing%20loss

[3] Onifade A , Katolo HW , Mookerjee S , Bhutta MF . Epidemiology of chronic suppurative otitis media: systematic review to estimate global prevalence. J Epidemiol Global Health. 2025;15(1):55. 10.1007/s44197-025-00396-9 PMC11968643 40178730

[4] Smith CDL , McMahon AD , Purkayastha M , et al. Head and neck cancer incidence is rising but the sociodemographic profile is unchanging: a population epidemiological study (2001‐2020). BJC Rep. 2024;2(1):71. 10.1038/s44276-024-00089-z PMC11408244 39301277

[5] Hu Q , Lv S , Wang X , Pan P , Gong W , Mei J . Global burden and future trends of head and neck cancer: a deep learning‐based analysis (1980‐2030). PLoS One. 2025;20(4):e0320184. 10.1371/journal.pone.0320184 PMC11981659 40203229

[6] Petrucci B , Okerosi S , Patterson RH , et al. The Global Otolaryngology‐Head and Neck Surgery Workforce. JAMA Otolaryngol Head Neck Surg. 2023;149(10):904‐911. 10.1001/jamaoto.2023.2339 PMC10472262 37651133

[7] Health RoRMo. 4X4 Reform: Executive Summary Towards Quadrupling the current workforce in 4 years (4x4); Uplifting Quantity and Quality of care. https://www.moh.gov.rw/index.php?eID=dumpFile&t=f&f=92859&token=3c26da47182b639efd749f9c6829633dc0fcada4

[8] Kim MK , Arsenault C , Atuyambe LM , Macwan'gi M , Kruk ME . Determinants of healthcare providers’ confidence in their clinical skills to deliver quality obstetric and newborn care in Uganda and Zambia. BMC Health Serv Res. 2020;20(1):539. 10.1186/s12913-020-05410-3 PMC7296707 32539737

[9] Harrell PL , Kearl GW , Reed EL , Grigsby DG , Caudill TS . Medical students’ confidence and the characteristics of their clinical experiences in a primary care clerkship. Acad Med. 1993;68(7):577‐579. 10.1097/00001888-199307000-00020 8323654

[10] Mughal Z , Gupta KK , Fruhstorfer B . Otolaryngology education in medical training: a literature review of current practice, challenges, and future directions. Ann Otol Rhinol Laryngol. 2025;134(10):783‐791. 10.1177/00034894251343373 40458060

[11] Matthews MR , Hicks KB , Shah RN , Buckmire R , DeMason CE . Promoting ENT‐erest: evaluating the effect of early, focused exposure to otolaryngology. OTO Open. 2025;9(4):e70167. 10.1002/oto2.70167 PMC12529453 41111614

[12] Groszkiewicz A , Patel H , Walden J , et al. Otolaryngology in medical education: hands‐on training at an SRFC community site improves medical student knowledge and skills. J Student‐Run Clin. 2024;10(1):1‐9. 10.59586/jsrc.v10i1.446

[13] Hu A , Sardesai MG , Meyer TK . A need for otolaryngology education among primary care providers. Med Educ Online. 2012;17:17350. 10.3402/meo.v17i0.17350 PMC3386554 22754276

[14] Domanski MC , Ashktorab S , Bielamowicz SA . Primary care perceptions of otolaryngology. Otolaryngol Head Neck Surg. 2010;143(3):337‐340. 10.1016/j.otohns.2010.05.012 20723768

[15] O'Brien DC , Squires LD , Robinson AD , Ramadan H , Diaz R . A multicenter, cross‐sectional assessment of otolaryngology knowledge among primary care trainees. Ann Otol Rhinol Laryngol. 2018;127(9):631‐636. 10.1177/0003489418784615 29952655

[16] World Health Organization. *Primary Ear and Hearing Care Training Manual*.

[17] Tsiga‐Ahmed F , Ahmed A . Effectiveness of an ear and hearing care training program for frontline health workers: a before and after study. Ann Afr Med. 2020;19(1):20‐25. 10.4103/aam.aam_9_19 PMC7189879 32174611

[18] Mulwafu W , Kuper H , Viste A , Goplen FK . Feasibility and acceptability of training community health workers in ear and hearing care in Malawi: a cluster randomised controlled trial. BMJ Open. 2017;7(10):e016457. 10.1136/bmjopen-2017-016457 PMC5652500 29025832

[19] Drobac PC , Basinga P , Condo J , et al. Comprehensive and integrated district health systems strengthening: the Rwanda Population Health Implementation and Training (PHIT) Partnership. BMC Health Serv Res. 2013;13(suppl 2):S5. 10.1186/1472-6963-13-S2-S5 PMC3668243 23819573

[20] Toniolo J , Ngoungou EB , Preux PM , Beloni P . Role and knowledge of nurses in the management of non‐communicable diseases in Africa: a scoping review. PLoS One. 2024;19(4):e0297165. 10.1371/journal.pone.0297165 PMC11025970 38635822

[21] Mhlongo EM , Lutge E , Adepeju L . The roles, responsibilities and perceptions of community health workers and ward‐based primary health care outreach teams: a scoping review. Glob Health Action. 2020;13(1):1806526. 10.1080/16549716.2020.1806526 PMC7782342 32892739

