# Supplementary material for: Opportunities to Improve Primary‐Level Otolaryngology Care: A Survey of Rwandan General Practitioners
Source: OTO Open. 2026 Oct 17;10(4):e70303. doi: 10.1002/oto2.70303 (PMC13676033; doi:10.1002/oto2.70303)
Supplement: Supplementary file 1 [file OTO2-10-e70303-s001.pdf]

ENT Baseline Needs Assessment for General Practitioners

Thank you so much for choosing to complete this survey! We hope to assess your thoughts and feelings regarding ENT education and treatment for primary health care workers. For each question, please take a moment to reflect on your experiences with ENT and head and neck patients. With your responses, we hope to craft meaningful curriculum to improve access to ENT education and enhance patients' health outcomes.

Study Consent

INFORMED CONSENT FORM

Principal Investigator: Dr. Gratien Tuyishimire
Name of Institution: University of Global Health Equity

Name of Project and Version: Otolaryngology Knowledge Assessment and Curriculum Development for Primary Care Providers in Rwanda

Part I: Information Sheet

My name is Dr.Gratien TUYISHIMIRE a Senior Consultant in OHNS and Adjunct faculty at University of Global Health Equity(UGHE).
This survey is about Otolaryngology Knowledge assessment and Curriculum Development for Primary Care providers in Rwanda. , and it is being done by the Global Otolaryngology-Head and Neck Surgery (OHNS) Initiative which is a non-profit organization focused on research in collaboration with UGHE. As a primary health worker, you are invited to participate and your participation is important and it will help us understand more about your otolaryngology knowledge and current ear and hearing care practices in our community.

Participating in this survey is your choice, and your answers will be kept private. The information you provide will be used for research, and it will be combined with responses from other health workers in your country. Once we remove your name from your responses, they may be shared with other researchers to improve global ear and hearing care without requiring additional informed consent.

By joining this survey, you confirm that you can use a computer, tablet, or smartphone and are okay spending around 15-20 minutes on the online survey. If you can't use technology devices our data collectors will reach out to you in person and bring hard copy questionnaire and will assist you to fill it and then they will record your answers on a tablet. You can skip any question or leave the study at any time without any impact on your professional standing with the Global OHNS Initiative or local ENT surgeons.

Purpose
It is possible the proportion of time allocated to theoretical and practical training in ENT for GPs and nurses is very limited and the CHWs don't have any training in ENT therefore the primary health care providers may lack basic knowledge and skills to address basic needs of patients with ENT diseases at primary healthcare level.
The aim of this study is to assess knowledge gaps and implement ENT-focused curriculum for the primary care workforce in Rwanda

Type of Research Intervention
A questionnaire to fill
Selection of Participants
You have been randomly selected considering our sample size and target group.

Voluntary Participation
Participating in this survey is your choice, and your answers will be kept private. You don't have to decide today. You can think about it and tell me what you decide later.
Protocol

You will be allowed to choose filling the online survey questionnaire or be handed a hard copy to fill by our data collectors who will be reach you to the nearest health center assist you to fill and then collect it. If you don't wish to answer some of the questions included in the questionnaire, you may skip them and move on to the next question. The information recorded is confidential, and no one else except Dr Gratien Tuyishimire and his research team will have access to your questionnaire. The hard copy questionnaires will be destroyed after your answers are uploaded to the password protected RedCap software.
Duration
Filling the questionnaire for this survey will take about 20minutes of your time .
Risks and Discomforts
The is no risk or discomforts associated with answering questions.

Benefits
There will be no immediate and direct benefit to you, but your participation is likely to help us find out more about the needs and develop a customized curriculum for our ENT primary care providers.
Incentives
By participating in the survey you will not be provided with any payment to take part in the research. However, for GPs who will participate in the in-person Training of Trainers their travel expenses will be covered .
Confidentiality:
We will not be sharing information about your son or daughter outside of the research team. The information that we collect from this research project will be kept confidential. Information that will be collected from the research will be put away and no-one but the researchers will be able to see it. Any information about you will have a number on it instead of the name. Only the researchers will know what your number is and we will keep that information in password protected RedCap

Sharing of Research Findings
You will be communicated about findings of this research. We will also publish the results in order that other interested people may learn from our research.
Right to refuse or withdraw
You may choose not to participate in this study. Choosing to participate or not will not affect either your relationship with ENT specialists of Rwanda.
You may stop participating in the survey at any time that you wish.

Who to Contact
If you have any questions you may ask them now or later, even after the study has started.
You can contact the Principal Investigator, Gratien Tuyishimire. Tel 0788800582 / Email: ishimegras@gmail.com. Or the other investigator Lt Col. Dr John Bukuru. Tel 0788754769/Email:johnbkrdr@gmail.com
This proposal has been reviewed and approved by the Rwanda National Ethic committee(RNEC), which is a committee whose task it is to make sure that research participants are protected from harm.
Should you wish to know more about RNEC or should you think your right is not respected during this research please reach out to the Chairman of the Committee: Dr Vedaste Ndahindwa on Tel:0788454613 or The Committee secretary Dr Marie Francoise Mukanyangezi on Tel: 0788672656.

PART II: Certificate of Consent

I have been asked to give consent to participate in this research study which will involve completing one questionnaire . I have been informed that there is no risks associated with completing the questionnaire. I am aware that there may be no benefit to me personally. I have been provided with the name of a researcher who can be easily contacted using the number I was given for that person.
I have read the foregoing information, or it has been read to me. I have had the opportunity to ask questions about it and any questions that I have asked have been answered to my satisfaction. I consent voluntarily to participate as a participant in this study and understand that I have the right to withdraw from the study at any time without any negative impact to me or my job.

Attachment: Study Informed consent form English .doc (0.12 MB)

If you consent to participate in this study, please provide us with the following information:

Full Name of Participant
* must provide value

Tel/WhatsApp Number
* must provide value

Email
* must provide value

Signature
* must provide value

Section I: Demographics

Age
* must provide value

Gender
* must provide value

Are you an intern doctor or a general practitioner?
* must provide value

- Intern Doctor
- General Practitioner
- Other

How many years have you been practicing medicine?
* must provide value

Where do you primarily work?
* must provide value

- Health Center
- District Hospital
- Provincial Referral
- Private Clinic
- Other

OPTIONAL - Please list any additional places you work

District

* must provide value

Section II: Training and Experience

How much ENT teaching did you receive in Medical School?

* must provide value

- None
- < 2 weeks
- 2 weeks to 1 month
- 1 to 2 months
- > 2 months
- Don't remember

Have you attended any ENT workshops or courses apart from at medical school?

* must provide value

- Yes
- No
- Don't remember

Do you think further emphasis of ENT training should be recommended for GP practice?

- Yes
- No

On an average week, how many patients do you see at the outpatient clinic with the following conditions? (You can make a weekly estimate based on what you see in a day or month).

Ear and hearing diseases (e.g. ear infections, mastoiditis, hearing loss)

* must provide value

(# of patients seen in a week, on average)

Diseases of the head and neck (e.g. cancer, thyroid diseases, salivary gland disease)

* must provide value

(# of patients seen in a week, on average)

Disease of the nose and sinus (e.g. rhinitis, sinusitis, polyps)

* must provide value

(# of patients seen in a week, on average)

Pediatric ENT (e.g. tonsilitis/ tonsillar hypertrophy, congenital neck masses, laryngomalacia)

* must provide value

(# of patients seen in a week, on average)

Laryngology (e.g. hoarseness of voice, laryngitis)

* must provide value

(# of patients seen in a week, on average)

Facial trauma (e.g. facial lacerations, facial fractures)

* must provide value

(# of patients seen in a week, on average)

Section III: Knowledge about recognition and management of ENT diseases

How many times in the last month have the following symptoms presented to you?

	0-5	6-14	15-25	> 25
**Sore throat** * must provide value				
**Dysphonia/hoarseness** * must provide value				
**Globus feeling in throat** * must provide value				
**Difficulty/noisy breathing** * must provide value				
**Hearing loss** * must provide value				
**Otalgia (ear pain)** * must provide value				
**Discharging ear** * must provide value				
**Tinnitus** * must provide value				
**Dizziness/Vertigo** * must provide value				
**Anosmia/Hyposmia** * must provide value				
**Rhinitis/Sinusitis** * must provide value				
**Facial palsy** * must provide value				
**Neck swelling/mass** * must provide value				
**Foreign body in ear** * must provide value				
**Foreign body in the nose** * must provide value				
**Foreign body in the throat/airway/esophagus** * must provide value				
**Epistaxis** * must provide value				

How confident are you in recognizing these common ENT conditions?

1 = very low confident
2 = low
3 = moderate
4 = confident
5 = very confident

	1 - very low confident	2 - low	3 - moderate	4 - confident	5 - very confident
**Pharyngitis/Laryngitis** * must provide value					
**Tonsillitis** * must provide value					
**Gastro-esophageal reflux disease** * must provide value					
**Infective and/or allergic rhinitis** * must provide value					
**Sinusitis** * must provide value					
**Epistaxis** * must provide value					
**Otitis media/Otitis media with effusion** * must provide value					

**Otitis externa**<br>* must provide value					
**Perforated tympanic membrane**<br>* must provide value	○	○	○	○	○
**Wax impaction in ear**<br>* must provide value	○	○	○	○	○
**Cholesteatoma**<br>* must provide value	○	○	○	○	○
**Benign paroxysmal positional vertigo**<br>* must provide value	○	○	○	○	○
**Head and neck masses**<br>* must provide value	○	○	○	○	○
**Laryngeal cancer**<br>* must provide value	○	○	○	○	○
**Adenoid/Tonsillar hypertrophy**<br>* must provide value	○	○	○	○	○
**Acute orbital complication of rhinosinusitis**<br>* must provide value	○	○	○	○	○
**Mastoiditis**<br>* must provide value	○	○	○	○	○
**Malignant otitis externa**<br>* must provide value	○	○	○	○	○
**Airway obstruction**<br>* must provide value	○	○	○	○	○
**Sudden sensorineural hearing loss**<br>* must provide value	○	○	○	○	○
**Facial nerve palsy/Bell's palsy**<br>* must provide value	○	○	○	○	○
**Deep space neck abscesses**<br>* must provide value	○	○	○	○	○
**Ludwig's Angina**<br>* must provide value	○	○	○	○	○
**Auricular hematoma/Perichondritis**<br>* must provide value	○	○	○	○	○
**Nasal septal hematoma/Abscess**<br>* must provide value	○	○	○	○	○
**Facial fractures**<br>* must provide value	○	○	○	○	○
**Foreign body in ears**<br>* must provide value	○	○	○	○	○
**Foreign body in nose**<br>* must provide value	○	○	○	○	○
**Foreign body in throat/esophagus**<br>* must provide value	○	○	○	○	○
**Foreign body in trachea or bronchus**<br>* must provide value	○	○	○	○	○
**Choanal atresia**<br>* must provide value	○	○	○	○	○

How confident are you in managing the following common ENT conditions?

1 = very low confident
2 = low
3 = moderate
4 = confident
5 = very confident

	1 - very low confident	2 - low	3 - moderate	4 - confident	5 - very confident
**Pharyngitis/Laryngitis**<br>* must provide value	○	○	○	○	○

**Tonsilitis**<br>* must provide value					
**Gastro-esophageal reflux disease**<br>* must provide value					
**Infective and/or allergic rhinitis**<br>* must provide value					
**Sinusitis**<br>* must provide value					
**Epistaxis**<br>* must provide value					
**Otitis media**<br>* must provide value					
**Otitis externa**<br>* must provide value					
**Wax impaction in ear**<br>* must provide value					
**Benign paroxysmal positional vertigo**<br>* must provide value					

How confident do you feel in providing initial or definitive management of the following ENT emergencies?

1 = very low confident
2 = low
3 = moderate
4 = confident
5 = very confident

	1 - very low confident	2 - low	3 - moderate	4 - confident	5 - very confident
**Orbital cellulitis/abscess**<br>* must provide value					
**Acute mastoiditis**<br>* must provide value					
**Airway obstruction**<br>* must provide value					
**Sudden sensorineural hearing loss**<br>* must provide value					
**Facial nerve palsy/Bell's palsy**<br>* must provide value					
**Deep space neck abscesses**<br>* must provide value					
**Ludwig's angina**<br>* must provide value					
**Auricular hematoma/Perichondritis**<br>* must provide value					
**Nasal septal hematoma/abscess/nasal fracture**<br>* must provide value					
**Foreign body in ears**<br>* must provide value					
**Foreign body in nose**<br>* must provide value					
**Foreign body in throat/esophagus**<br>* must provide value					
**Foreign body in trachea or bronchus**<br>* must provide value					
**Malignant otitis externa**<br>* must provide value					
**Choanal atresia**<br>* must provide value					

How confident do you feel performing the following ENT procedures?

1 = very low confident
2 = low
3 = moderate
4 = confident
5 = very confident

	1 - very low confident	2 - low	3 - moderate	4 - confident	5 - very confident
**Recognizing the normal ear, nose, and throat anatomy** * must provide value	◯	◯	◯	◯	◯
**Otoscopy** * must provide value	◯	◯	◯	◯	◯
**Anterior rhinoscopy** * must provide value	◯	◯	◯	◯	◯
**Neck examination** * must provide value	◯	◯	◯	◯	◯
**Tuning fork test** * must provide value	◯	◯	◯	◯	◯
**Nasal foreign body removal** * must provide value	◯	◯	◯	◯	◯
**Ear foreign body removal** * must provide value	◯	◯	◯	◯	◯
**Ear syringing** * must provide value	◯	◯	◯	◯	◯
**Anterior nasal packing for epistaxis** * must provide value	◯	◯	◯	◯	◯
**Posterior nasal packing for epistaxis** * must provide value	◯	◯	◯	◯	◯
**Tracheostomy care (suctioning, changing)** * must provide value	◯	◯	◯	◯	◯
**Incision and drainage of superficial neck abscesses** * must provide value	◯	◯	◯	◯	◯
**Lymph node biopsy** * must provide value	◯	◯	◯	◯	◯
**Repair of simple facial lacerations** * must provide value	◯	◯	◯	◯	◯

If there are other ENT conditions which you would like more training in, please describe:

Section IV: Challenges and gaps encountered by GPs in managing ENT diseases

What gaps do you think GPs encounter in Rwanda while managing ENT diseases? (Pick all that apply)
* must provide value

- ☐ Limited training/knowledge/skills
- ☐ Limited diagnostic tools to work up ENT diseases
- ☐ Limited equipment and materials to treat ENT conditions
- ☐ Delayed patient presentation
- ☐ Challenges with referring patients to higher level facilities
- ☐ Other

How often do you experience the following?

	All the time	Often	Sometimes	Rarely	Never
**How often do you see patient/family refusal to transfer to an ENT specialist?** * must provide value	○	○	○	○	○
**How often do you see difficulties in getting an appointment to an ENT specialist?** * must provide value	○	○	○	○	○
**How often do you see delayed transfer due to bed availability at the accident and emergency department?** * must provide value	○	○	○	○	○
**How often do you see preventable complications resulting from delayed transfer?** * must provide value	○	○	○	○	○

Educational Opportunities

With these survey results, we are planning to create an in-person education/training opportunity with hands-on activities and case-based learning on Friday April 4th, 2025 and Saturday April 5th, 2025. Do these dates work for you?

* must provide value

○ Yes
○ No

Closing Question

In the future, if we need to reach back out for additional questions, would you be willing to be contacted?

* must provide value

○ Yes
○ No

Submit
